# Health literacy of vocational and university students in the allied health professions in Germany—a cross-sectional study

**DOI:** 10.3389/fpubh.2025.1711608

**Published:** 2025-12-04

**Authors:** Angela Gerhard, Madeleine Beck, Simone Goedeke-Boch, Karin Kohlstedt, Ruth Nobis-Bosch, Tobias Braun

**Affiliations:** 1 Department of Health HSD Hochschule Döpfer, Cologne, Germany; 2 Department of Speech and Language Therapy, Trainig Academy for Health Professions University Hospital RWTH Aachen, Aachen, Germany

**Keywords:** health literacy, digital health literacy, health professional, allied health professions, cross-sectional study

## Abstract

**Background:**

Evidence indicates that many vocational and university students in the allied health professions in Germany lack sufficient health literacy. However, strengthening health literacy is particularly important for this group, as these individuals play a key role in conveying health information. The objective of this study is to describe the levels of general and digital health literacy among vocational and university students in the allied health professions in Germany and to explore possible influencing factors.

**Methods:**

This exploratory, cross-sectional study was conducted through an online survey among vocational and university students in allied health professions in Germany. Data were collected using standardized questionnaires on general and digital health literacy. The survey was conducted in 12/2022 and included also socio-demographic data and health behavior. Various socio-demographic characteristics were examined for their association with health literacy through regression analyses.

**Results:**

Data from 655 individuals were included in the analyses. On average, general health literacy was 67.4% (100% = maximum health literacy), with 53.4% participants displaying high general health literacy. The average digital health literacy was 60.9% (44.3% high digital health literacy). The only statistically significant indicator for higher general health literacy was male gender. In contrast, for digital health literacy, male gender, higher self-efficacy, higher alcohol consumption, and older age were associated with higher digital health literacy.

**Conclusion:**

In this study, vocational and university students in the allied health professions showed higher health literacy levels compared to the general population in Germany. To improve competence, content related to health literacy could be systematically integrated into curricula.

## Introduction

Health literacy is defined as people’s knowledge, motivation and competences to access, understand, appraise, and apply health information to make judgments and take decisions in everyday life concerning healthcare, disease prevention and health promotion to maintain or improve quality of life (1, 2). Health literacy is regarded as a key resource for the prevention, early detection, and management of illness and injury (3, 4). Individuals with higher health literacy are generally more capable of navigating healthcare systems. This is reflected in more effective communication with physicians, nurses, therapists, and other healthcare professionals, which in turn facilitates informed decision-making regarding their care (1). Low health literacy is associated with increased mortality, poorer physical and mental health status, higher medication use, and a greater risk of disease chronicity (1, 5).

In addition to general health literacy, other dimensions have been identified, such as digital health literacy, for which a universally accepted international definition is still lacking (6). Digital health literacy can be understood as the ability to appropriately access, interpret, and use digital health information (6, 7). The digital health literacy encompasses individual, social, and technical skills and resources required to search for, find, understand, appraise, and apply digitally available health information (1, 8).

Current studies show that at least half of the German population exhibits limited health literacy. For example, 54% of the population in Germany have limited general health literacy (5) and 52% have limited digital health literacy (9). National and international studies identified several factors associated with low health literacy. These include migration background, low education, low socioeconomic status, chronic illness, and older age (65+ years) (5, 9).

Healthcare professionals play a vital role in the promotion and communication of health literacy (10, 11). This group includes not only medical doctors (e.g., general practitioners and specialists), but also nurses, therapists, paramedics, and midwives (12). Due to their professional education and training, healthcare professionals typically possess greater knowledge of health and health promotion than the general population. They are therefore well-positioned to foster and improve health literacy among service users, such as individuals with chronic conditions, people in need of care, and pregnant women (11, 13–15). However, healthcare professionals often do not (sufficiently) take on the task of promoting their patients’ health literacy. This could be because they are not adequately sensitized to this topic, and health literacy is usually not explicitly included in the training curricula, or they themselves do not have sufficient health literacy (1, 16). For instance, a study by Janson and Rathmann (17) found that only 41% of outpatient nurses demonstrated “sufficient” health literacy, while 13% were rated as having “problematic” health literacy. Kädling (18) reported that 77% of professional nurses exhibited low health literacy.

The health literacy of future healthcare professionals should therefore be taught and strengthened during their educational training. Initial evidence suggests, however, that a considerable proportion of healthcare students and trainees in Germany also exhibit insufficient health literacy. For example, Reick et al. (19) and Simon et al. (20) found that only 30% of students at a university of applied sciences or prospective healthcare professionals had sufficient or excellent health literacy skills.

The primary aim of the present study is to assess and describe the levels of general and digital health literacy among students and trainees in the allied health professions in Germany. Furthermore, the study seeks to explore which factors influence the development of health literacy.

## Methods

### Study design and procedure

This explorative, quantitative cross-sectional study (online survey using SoSci Survey (SoSci Survey GmbH, 2022)) among trainees and students of the allied health professions in Germany was reviewed and approved by the ethics committee of HSD Hochschule Döpfer GmbH (Number: BEth_48_22). Participants were informed about the study in the introductory section of the survey, and informed consent was obtained electronically before participation. Participation in the study was voluntary and available from December 5 to December 31, 2022, via a public access link. To encourage participation, ten gift vouchers of €10 each were raffled among respondents. The study was not registered, and no study protocol was published before the study was conducted. Reporting followed the STROBE guidelines (21), CHERRIES checklist criteria (22), and the SAMPL guidelines (23).

### Participants and recruitment

All trainees and students in the allied health professions (nursing, emergency services, physiotherapy, occupational therapy, speech and language therapy, midwifery or medical-technical professions) who were undergoing a training of at least 3 years or who were studying at a German vocational college or university and were aged 18 or over at the time of the survey were eligible. People who already had another vocational or academic qualification (“second-chance education”) were also able to take part in the study, provided they had no more than 2 years of professional experience in this occupation.

Participants were recruited (convenience sample) via three ways: (1) Unsystematically selected vocational schools and universities of health and therapy sciences throughout Germany were contacted with a request to forward the survey to trainees and students. (2) Trainees and students from the project team’s working environment (teachers at schools and universities for healthcare professions) were invited to participate in the study. This recruitment took place in groups (cohorts/classes) and not at the individual level. (3) The survey was disseminated via professional associations and interest groups (snowball system).

### Variables

The variables collected in this study on the socio-demographic characteristics of the participants, their educational situation and their own health and health-related behavior are listed in the Supplementary material 1, including operationalization, response options and sources. Where possible, the socio-demographic variables and potential influencing factors were collected using standardized items from the GEDA-EHIS Survey (24) or taken from other (validated) questionnaires/surveys. General health literacy and digital health literacy were assessed using standardized questionnaires with evidence for sufficient measurement properties (25).

### Data sources and measurement (online survey)

General health literacy was assessed using the validated HLS19-Q16-DE instrument (26). This questionnaire was developed as part of the European Health Literacy Survey (HLS-EU) (27). We used a short version which is based on a questionnaire with 47 items that measures the four dimensions of general health literacy (accessing, understanding, evaluating, and applying health information) in the areas of disease prevention, health promotion and health care. The 16 items of the short version refer to various tasks and activities related to health care, disease prevention or health promotion (24). The response options allowed for a subjective assessment of the corresponding task or activity with the options “very easy” (4), “easy” (3), “difficult “(2), “very difficult” (1) and “do not know/no answer.” The HLS19-Q16-DE score was calculated as the percentage (ranging from 0 to 100) of items with valid responses that were answered with “very easy” or “easy,” provided that at least 80% of the items (i.e., ≥ 13 items) contained valid responses.

Digital health literacy was assessed using HLS19-DIGI-DE, which is a questionnaire with a total of 10 items, 8 of which relate to self-assessed difficulties in finding, understanding, assessing and using digital health information (26). Based on the 10 items, the digital health literacy score was calculated as the percentage (ranging from 0 to 100) of items with valid answers that were answered with “very easy” or “easy.” Here, too, at least 80% of the items (i.e., ≥ 8 items) had to contain valid answers.

The values of the HLS19-Q16-DE and HLS19-DIGI-DE were each reported as a continuous variable from 0 to 100%, with higher values indicating more pronounced health literacy. Health literacy and digital health literacy levels were categorized as inadequate (0–50%), problematic (>50–66%), sufficient (>66–84%), and excellent (84–100%) (28). In addition, a dichotomous classification into “low” (0–66.67%) and “high” (≥66.68–100%) general or digital health literacy was used (28). The HLS-EU Consortium holds the rights to both instruments. Permission to use version 6 of the German translations was obtained.

The online survey consisted of 5 sections over 11 pages (Supplementary material 2).

• Section 1 (Pages 1–3): Socio-demographic data and information on education.

• Section 2 (Pages 4–7): Health status and health-related behaviors.

• Section 3 (Page 8): General health literacy (HLS19-Q16-DE).

• Section 4 (Page 9): Digital health literacy (HLS19-DIGI-DE).

• Section 5 (Pages 10–11): Closing information.

If participants attempted to skip a question, a prompt appeared. However, it was possible to continue without providing an answer.

The survey underwent pilot testing with 12 health professionals from a Master’s program in medical education (with various backgrounds), and revisions were made based on their feedback. The final version of the questionnaire (including screenshots) is available in the Mendeley Data Repository of this research project (doi: 10.17632/xhd86y2vwc.1).

### Bias

It is possible that individual study participants did not complete the survey carefully [so-called “careless response” bias (29, 30)]. To avoid this risk of information bias, all study participants were given sufficient time to complete the survey. Where possible, the socio-demographic variables and potential influencing factors were collected using standardized items from the GEDA-EHIS survey (24) or taken from other (validated) questionnaires/surveys (Supplementary material 1). Standardized, validated questionnaires were also used to assess general and digital health literacy (25).

The risk of selection bias arose due to unsystematic recruitment, mainly through the project team’s professional network. To counteract this, a broad recruitment strategy was implemented, including outreach to institutions and the use of snowball sampling.

### Data collection and analysis

No formal sample size calculation was performed due to the convenience sampling strategy. However, a minimum of 500 participants was targeted according, as recommended (31). After data collection, cases that did not meet the inclusion criteria (e.g., under 18 years of age) or had incomplete HLS19-Q16-DE data (<80% answered) were excluded.

Socio-demographic variables were analyzed descriptively. Cases with missing data were excluded case-wise. General and digital health literacy scores were reported both categorically (inadequate, problematic, sufficient, excellent) and dichotomously (low vs. high) (28). For exploratory analysis, participants were dichotomized based on health literacy levels: Low: 0–66.67%; High: ≥66.68–100%.

In univariable regression analyses, based on assumptions of possible associations (32), 21 different characteristics of the participants (predictors) were first analyzed for their correlations with general health literacy or digital health literacy, such as gender, profession, type and phase of education and health behavior. Metric data was summarized in logical categories so that each characteristic had a size of at least 10 participants (33). For characteristics with more than two attributes, either the attribute with the most participants was selected as the reference or the attribute that we considered most likely to correspond to clinical practice. The results of the regression analysis were expressed as odds ratios (OR) with 95% confidence intervals (95% CI).

The relationship between the characteristics (predictors) and health literacy was then examined further based on the results of the univariable regression analyses. For this purpose, the variables that were statistically significant at the 0.25 level in the univariable models were included simultaneously in the regression models. This threshold is based on recommendations to reduce the risk of selection bias (34).

Data were analyzed using SPSS (Version 25.0, IBM Corp.) and JASP (Version 0.16, University of Amsterdam). Statistical significance was set at *p* < 0.05.

## Results

**Figure 1:**
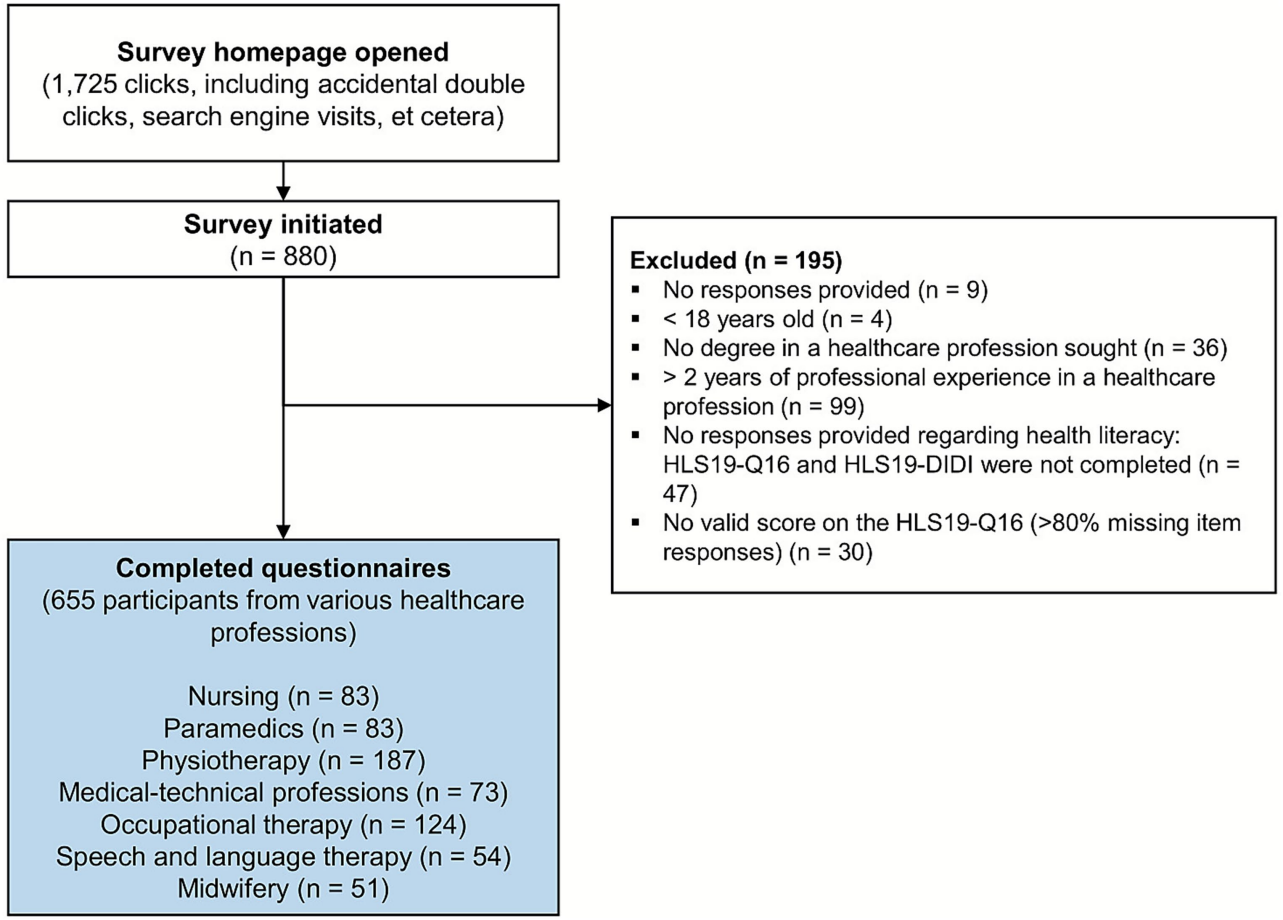
Flow chart.

The survey was initiated 880 times during the survey period. After excluding incomplete and invalid data sets, the data of 655 study participants were analyzed (flow chart in Figure 1). The socio-demographic characteristics of the sample are shown in Table 1 (additional information in Supplementary material 2). The average age of the participants was 22.9 ± 4.9 years and 77% were female.

**Table 1:** Characteristics and health behavior of participants (*n* = 655).

Age in years (*n* = 655), mean ± SD (range)	22.9 ± 4.9 (18–57)
Health status (self-reported, *n* = 653), scale: 0–100, mean ± SD (range)	74.9 ± 15.3 (6–100)
Self-efficacy (*n* = 655), scale: 0–12, median (IQR)	9 (8–10)

	*n*	Percent (%)
Gender (*n* = 650)		
Male	145	22.3
Female	497	76.5
Diverse	8	1.2
Migration background (*n* = 646)		
Yes	89	13.8
No	557	86.2
Highest educational degree (*n* = 655)		
Lower secondary school	2	0.3
Upper secondary school	133	20.3
University of applied sciences entrance qualification	118	18
University entrance qualification	350	53.4
Bachelor	33	5.0
Master	12	1.8
Other (please report)	7	1.1
Completed medical training/studies (*n* = 655)		
Yes (<2 years of professional experience)	58	8.9
No	597	91.1
Type of training (*n* = 655)		
Vocational school training	527	80.5
Dual studies	67	10.2
University studies	61	9.3
Degree sought (*n* = 655)		
State examination	493	75.3
Bachelor	148	22.6
Master	14	2.1
Training year (*n* = 526)		
1. Year	189	35.9
2. Year	177	33.7
3. Year	158	30.0
4. Year or more	2	0.4
Study phase (semester; *n* = 129)		
1. or 2. Semester	33	25.6
3. or 4. Semester	54	41.9
5. or 6. Semester	18	14.0
7. or 8. Semester	19	14.7
9. Semester or more	5	3.9
Socio-demographic status (self-reported, *n* = 641)		
High	89	13.9
Medium	446	69.6
Low	106	16.5
Quality of life (*n* = 655)		
Very bad	0	0
Bad	14	2.1
Sufficent	101	15.4
Satisfactory	197	30.1
Good	299	45.6
Very good	44	6.7
Mindfulness towards own health (*n* = 655)		
Very much	40	6.1
Much	226	34.5
Moderate	336	51.3
Less	52	7.9
Not at all	1	0.2
Physical activity (*n* = 655)		
Never	66	10.1
1×/month	101	15.4
1×/week	176	26.9
2-3×/week	214	32.7
4-5×/week	78	11.9
Daily	20	3.1
Healthy diet (*n* = 636)		
Rarely	92	14.5
1×/week	75	11.8
2-3×/week	323	50.8
Every day	146	23.0
Social support (number of support persons; *n* = 655)		
None	8	1.2
1–2	166	25.3
3–5	346	52.8
6 or more	135	20.6
Tobacco consumption (*n* = 650)		
Yes. Daily	87	13.4
Yes. Occasionally	69	10.6
No. Not anymore	67	10.3
Never smoked	427	65.7
Alcohol consumption (*n* = 638)		
Daily	1	0.2
Several times a week	34	5.3
Once a week	113	17.7
At least once a month	282	44.2
I do not drink alcohol	208	32.6
Chronic disease (*n* = 648)		
Yes	213	32.9
No	435	67.1
Mental health disorder (*n* = 636)		
Yes	180	28.3
No	456	71.7
Regular medication use (≥3 times/month; *n* = 654)		
Yes	293	44.8
No	361	55.2

SD, standard deviation; IQR, interquartile range. Values are absolute numbers (%) or as stated. Totals may vary due to missing data for individual variables. Missing values = “not specified”.

**Figure 2:**
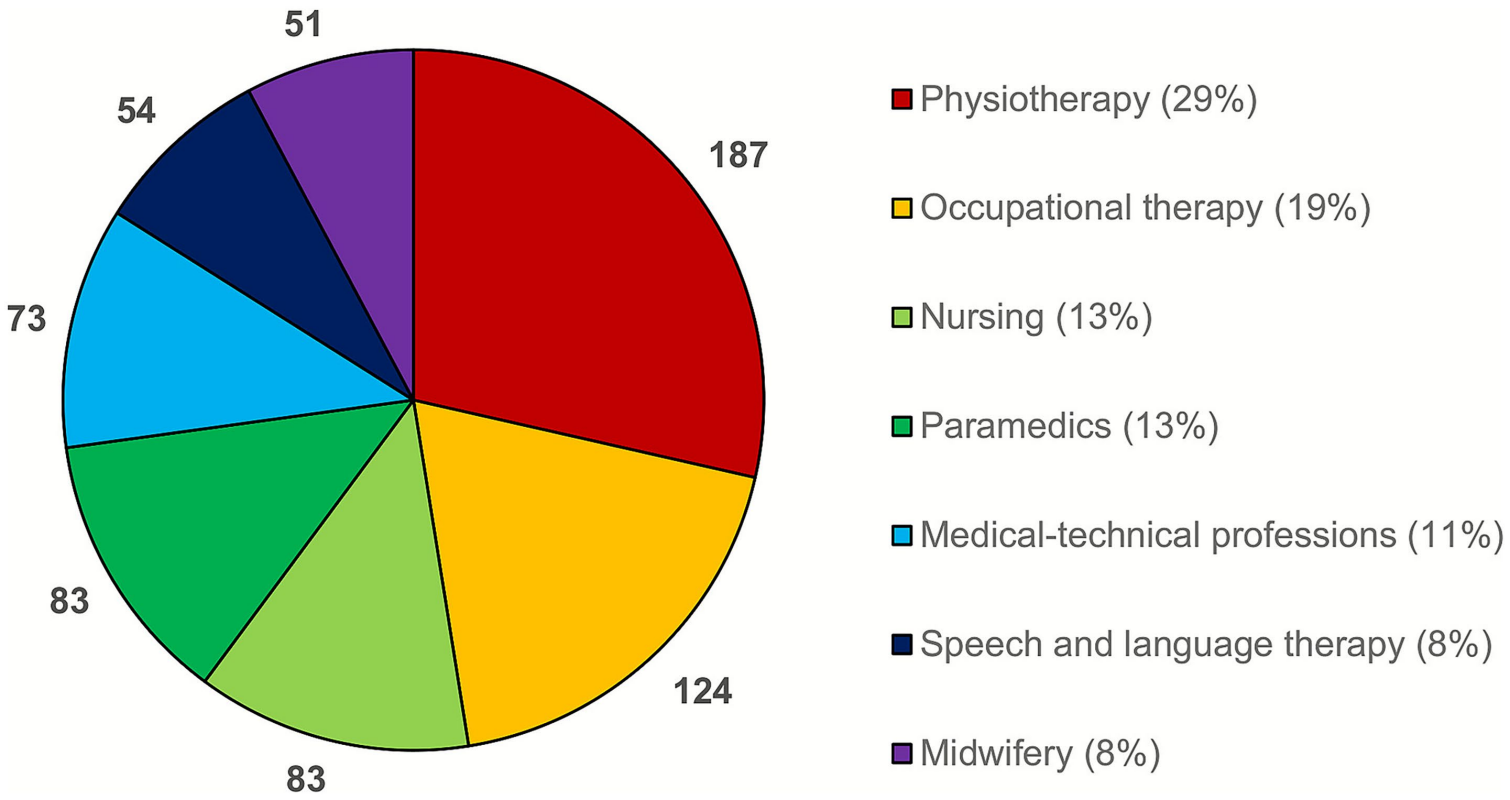
Composition of the sample by profession (*n* = 655).

Most respondents were trainees or students in physiotherapy (187/655, 29%), occupational therapy (124/655, 19%), or nursing and emergency medical services (83/655 each, 13%; Figure 2). Most participants stated a place of residence in North Rhine-Westphalia (239/655, 36%), Baden-Württemberg (182/655, 28%) or Hesse (121/655, 18%). The absolute and relative distribution of participants across the 16 federal states in Germany is shown in the Supplementary material 3.

**Figure 3:**
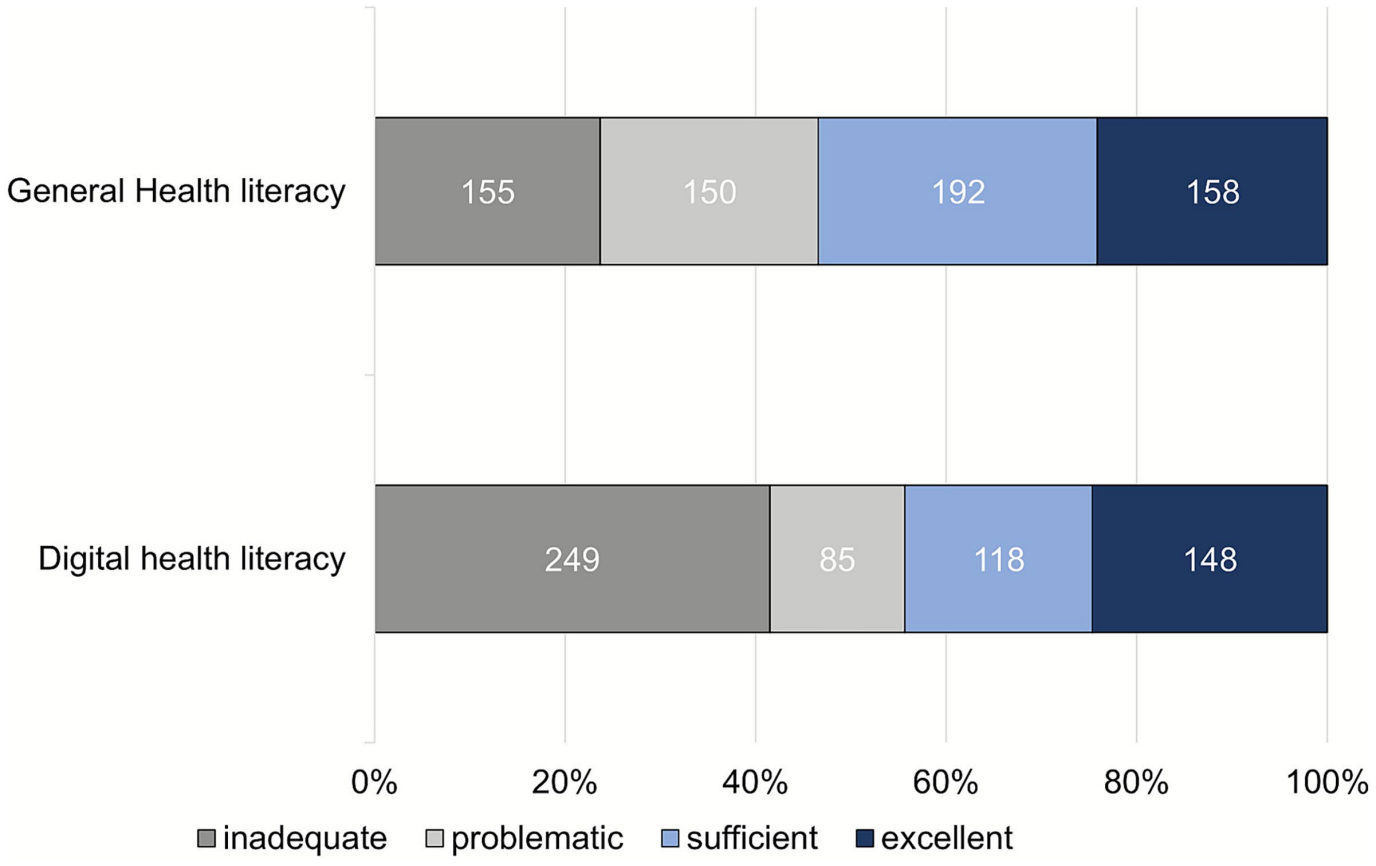
Level of general (*n* = 655) and digital (*n* = 600) health literacy.

All 655 participants completed the questionnaire on general health literacy. The average health literacy score was 67.4 ± 21.1% (range: 0–100; 95% CI: 65.8–69.0). The assessment of the level of health literacy based on the 4 defined categories (Figure 3) shows that 53.4% (350/655) of the participants have a high level of general health literacy. The response behavior of the participants at the item level is shown in the figure in the Supplementary material 4.

The following 3 items were most frequently rated as “difficult/very difficult”: “Assess whether a second medical opinion should be sought” (HLSQ16 item 05); “Find information on dealing with mental health problems” (HLSQ16 item 08); “Assess the trustworthiness of information on health risks in the media” (HLSQ16 item 11).

Univariable and multivariable logistic regression models for general health literacy (Table 2) indicated that male gender was the only statistically significant predictor of higher general health literacy (OR: 2.05; 95% CI: 1.31–3.21).

**Table 2:** Associations between participants’ socio-demographic characteristics and health behavior (predictors) and general health literacy (outcome), analyzed using univariable and multivariable logistic regression models (*n* = 655).

Predator variable	High health literacy		Univariable logistic regression		Multivariable logistic regression	
	*n*	Percent	OR [95% CI]	*p* value	OR [95% CI]	*P* value
Gender						
Female	245/497	49.3	Reference		Reference	
Male	98/145	67.6	2.15 [1.45–3.17]	<0.001	2.05 [1.31–3.21]	<0.01
Age						
18–21 years	152/323	47.1	Reference		Reference	
≥22 years	198/322	61.5	1.67 [1.22–2.27]	0.001	1.41 [0.99–2.01]	0.05
Migration background					Not included	
No	293/557	52.6	Reference			
Yes	52/89	58.4	1.27 [0.81–1.99]	0.31		
Education						
Lower/upper secondary school	64/135	47.4	Reference		Reference	
University entrance qualification	281/513	54.8	1.34 [0.92–1.97]	0.13	1.39 [0.90–2.14]	0.14
Completed training in the medical field					Not included	
No	315/597	52.8	Reference			
Yes (<2 years of experience)	35/58	60.3	1.36 [0.79–2.36]	0.27		
Profession					Not included	
Nursing	46/83	55.4	Reference			
Paramedics	50/83	60.2	1.22 [0.66–2.26]	0.53		
Physiotherapy	100/187	53.5	0.93 [0.55–1.55]	0.77		
Medical-technical professions	41/73	56.2	1.03 [0.55–1.94]	0.93		
Occupational therapy	60/124	48.4	0.75 [0.43–1.32]	0.32		
Speech and language therapy	27/54	50.0	0.80 [0.41–1.60]	0.53		
Midwifery	26/51	50.1	0.84 [0.42–1.68]	0.62		
Type of education					Not included	
Training	277/527	52.6	Reference			
(Dual) university studies	73/128	57.0	1.20 [0.81–1.77]	0.36		
Stage of education					Not included	
1. Year/1.–2. semester	109/222	49.1	Reference		Reference	
2. Year/3.–4. semester	124/231	53.7	1.20 [0.83–1.74]	0.33	1.27 [0.84–1.92]	0.26
3. Year or more/ 5. semester or more	117/202	57.9	1.43 [0.97–2.09]	0.07	1.40 [0.90–2.16]	0.14
Socio-demographic status					Not included	
Low	60/106	56.6	Reference			
Medium	49/89	55.1	0.85 [0.55–1.30]	0.44		
High	234/446	52.5	0.94 [0.53–1.66]	0.83		
Quality of life						
Low	150/312	48.1	Reference		Reference	
High	200/343	58.3	1.51 [1.11–2.06]	0.01	1.35 [0.93–1.96]	0.12
Health status						
Low	149/300	49.7	Reference		Reference	
High	200/353	56.7	1.33 [0.97–1.81]	0.08	1.03 [0.71–1.49]	0.90
Mindfulness towards own health						
Less	199/389	51.2	Reference		Reference	
Strong	151/266	56.8	1.25 [0.92–1.72]	0.16	1.02 [0.69–1.51]	0.93
Self-efficacy						
Low	240/475	50.5	Reference		Reference	
High	110/180	61.1	1.54 [1.09–2.18]	0.02	1.41 [0.95–2.10]	0.09
Physical activity						
Never/rarely	79/167	47.3	Reference		Reference	
Frequently	100/176	56.8	1.47 [0.96–2.24]	0.08	1.46 [0.91–2.35]	0.12
Often	171/312	54.8	1.35 [0.93–1.97]	0.12	1.22 [0.78–1.90]	0.38
Diet						
Bad	81/167	48.5	Reference		Reference	
Good	260/469	55.4	1.32 [0.93–1.88]	0.12	1.03 [0.68–1.57]	0.89
Social support						
Low	80/147	54.4	Reference		Reference	
High	270/481	56.1	1.50 [1.06–2.13]	0.02	1.20 [0.80–1.79]	0.38
Tabacco					Not included	
No	266/494	53.8	Reference			
Yes	83/156	53.2	0.98 [0.68–1.40]	0.89		
Alcohol						
Little	320/603	53.1	Reference		Reference	
Much	23/35	65.7	1.70 [0.83–3.47]	0.15	1.42 [0.66–3.06]	0.37
Chronic disease						
No	244/435	56.1	Reference		Reference	
Yes	103/213	48.4	0.73 [0.53–1.02]	0.06	0.77 [0.53–1.13]	0.19
Mental health disorder					Not included	
No	249/456	54.6	Reference			
Yes	93/180	51.7	0.89 [0.63–1.26]	0.50		
Regular medication use						
No	204/361	56.5	Reference		Reference	
Yes	146/293	49.8	0.76 [0.56–1.04]	0.09	1.06 [0.73–1.53]	0.76

OR, odds ratio; CI, confidence interval.

Model parameters of the multivariable regression model: Pseudo coefficient of determination *R*^2^ = 0.05 (McFadden), 0.09 (Nagelkerke), and 0.07 (Cox and Snell); Chi^2^ (576) = 42.97, *p* < 0.001.

Evaluable data sets from 600 participants were available for digital health literacy. The average digital health literacy was 60.9 ± 28.0% (range: 0–100; 95% CI: 58.7–63.2), with 44.3% (266/600) of participants demonstrating a high level of general health literacy (Figure 3). The figure in the Supplementary material 5 shows the response behavior of the participants at the item level. The following 3 items were most frequently rated as “difficult/very difficult”: “Find exactly the information you are looking for” (HLS19-DIGI-DE item 2.2); “Judge how trustworthy the information found is” (HLS19-DIGI-DE item 2.4); “Judge whether there are commercial interests behind the information provided” (HLS19-DIGI-DE item 2.5).

The results of the univariable and multivariable logistic regression models for digital health literacy are shown in Table 3. There was a statistically significant correlation between more pronounced digital health literacy and male gender (OR: 1.98; 95% CI: 1.26–3.11), older age (OR: 1.67; 95% CI: 1.15–2.41), higher self-efficacy (OR: 1.53; 95% CI: 1.03–2.27) and higher alcohol consumption (OR: 2.38; 95% CI: 1.00–5.63).

**Table 3:** Associations between participants’ socio-demographic characteristics and health behavior (predictors) and digital health literacy (outcome), analyzed using univariable and multivariable logistic regression models (*n* = 600).

Predator variable	High digital health literacy		Univariable logistic regression		Multivariable logistic regression	
	*n*	Percent	OR [95% CI]	*P* value	OR [95% CI]	*P* value
Gender						
Female	178/453	39.3	Reference		Reference	
Male	82/135	60.7	2.39 [1.61–3.54]	<0.001	1.98 [1.26–3.11]	0.003
Age						
18–21 years	103/290	35.5	Reference		Reference	
≥22 years	163/310	52.6	2.01 [1.45–2.79]	<0.001	1.67 [1.15–2.41]	0.007
Migration background					Not included	
No	228/509	44.8	Reference			
Yes	37/83	44.6	0.99 [0.62–1.58]	0.97		
Education					Not included	
Lower/upper secondary school	56/120	46.7	Reference			
University entrance qualification	207/473	43.8	0.89 [0.60–1.33]	0.57		
Completed training in the medical field						
No	234/547	42.8	Reference		Reference	
Yes (<2 years of experience)	32/53	60.4	2.04 [1.15–3.63]	0.02	1.55 [0.83–2.87]	0.17
Profession						
Nursing	39/77	50.6	Reference		Reference	
Paramedics	39/74	52.7	1.09 [0.57–2.06]	0.80	0.95 [0.47–1.95]	0.90
Physiotherapy	69/177	39.0	0.62 [0.36–1.07]	0.09	0.61 [0.34–1.12]	0.11
Medical-technical professions	28/66	42.4	0.72 [0.37–1.39]	0.33	0.87 [0.42–1.78]	0.70
Occupational therapy	44/108	40.7	0.67 [0.37–1.21]	0.18	0.81 [0.43–1.54]	0.52
Speech and language therapy	25/51	49.0	0.94 [0.46–1.90]	0.86	1.29 [0.60–2.79]	0.51
Midwifery	22/45	46.8	0.86 [0.42–1.77]	0.68	1.08 [0.50–2.34]	0.85
Type of education					Not included	
Training	211/479	44.1	Reference			
(Dual) university studies	55/121	45.5	1.06 [0.71–1.58]	0.78		
Stage of education						
1. Year/1.–2. semester	79/192	41.1	Reference		Reference	
2. Year/3.–4. semester	94/220	42.7	1.07 [0.72–1.58]	0.75	1.05 [0.68–1.63]	0.82
3. Year or more/ 5. Semester or more	93/188	49.5	1.40 [0.93–2.10]	0.10	1.14 [0.72–1.79]	0.58
Socioeconomic status					Not included	
Low	48/100	48.0	Reference			
Medium	182/405	44.9	0.88 [0.57–1.37]	0.58		
High	34/84	40.5	0.74 [0.41–1.32]	0.31		
Quality of life					Not included	
Low	122/283	43.1	Reference			
High	144/317	45.4	1.10 [0.80–1.52]	0.57		
Health status					Not included	
Low	120/273	44.0	Reference			
High	145/325	44.6	1.03 [0.74–1.42]	0.87		
Mindfulness towards own health						
Less	143/346	41.3	Reference		Reference	
Strong	123/254	48.4	1.33 [0.96–1.85]	0.08	1.30 [0.88–1.92]	0.19
Self-efficacy						
Low	172/429	40.1	Reference		Reference	
High	94/171	55.0	1.82 [1.28–2.61]	<0.001	1.53 [1.03–2.27]	0.03
Physical activity						
Never/rarely	57/146	39.0	Reference		Reference	
Frequently	74/161	46.0	1.33 [0.84–2.09]	0.22	1.37 [0.83–2.24]	0.22
Often	135/293	46.1	1.33 [0.89–2.00]	0.16	1.27 [0.79–2.03]	0.33
Diet					Not included	
Bad	63/151	41.7	Reference			
Good	199/434	45.9	1.18 [0.81–1.72]	0.38		
Social support					Not included	
Low	69/167	41.3	Reference			
High	197/433	45.5	1.19 [0.83–1.70]	0.36		
Tabacco					Not included	
No	192/460	41.7	Reference		Reference	
Yes	73/137	53.3	1.59 [1.09–2.34]	0.02	1.48 [0.96–2.29]	0.08
Alcohol						
Little	243/554	43.9	Reference		Reference	
Much	20/29	69.0	2.84 [1.27–6.36]	0.01	2.38 [1.00–5.63]	0.049
Chronic disease					Not included	
No	173/396	43.7	Reference			
Yes	91/198	46.0	1.10 [0.78–1.55]	0.60		
Mental health disorder					Not included	
No	185/415	44.6	Reference			
Yes	77/168	45.8	1.05 [0.73–1.51]	0.78		
Regular medication use					Not included	
No	148/326	45.4	Reference			
Yes	117/273	42.9	0.90 [0.65–1.25]	0.53		

OR, odds ratio; CI, confidence interval.

Model parameters of the multivariable regression model: Pseudo coefficient of determination *R*^2^ = 0.07 (McFadden), 0.13 (Nagelkerke), and 0.09 (Cox and Snell); Chi^2^ (551) = 56.34, *p* < 0.001.

**Figure 4:**
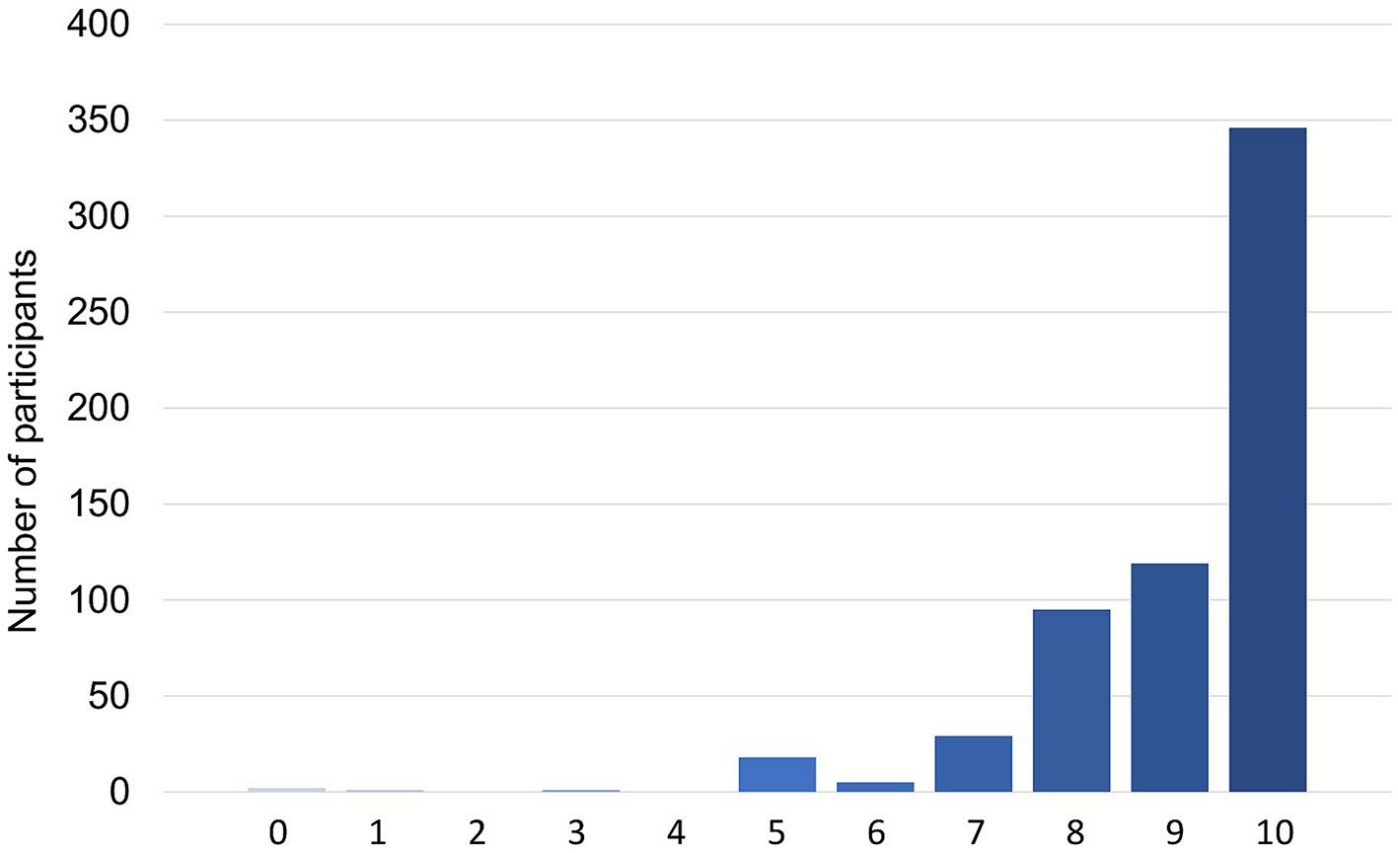
Importance of health literacy for healthcare professionals (616 participants; 39 not responding); scale: 0 = “no importance” to 10 = “very high importance”.

A total of 91% (560/616) of participants stated that health literacy is of high importance (scores 8–10) for healthcare professionals (Figure 4).

## Discussion

The findings of this study indicate that students and trainees in the allied health professions in Germany exhibit a higher level of general health literacy (67.4%) compared to similar target populations in previous research. Löwe et al. (35) found that during the COVID-19 pandemic, only 42.7% of students in Germany had sufficient health literacy, while 41.5% were classified as problematic and 15.8% as inadequate. Schultes (36) reported an average health literacy of 33.5% among German students. Similarly, Reick et al. (19) reported that only 2.4% of students at a university of health sciences demonstrated excellent health literacy, with 20.5% inadequate, 48.8% problematic, and 28.4% sufficient. Simon et al. (20) also reported that only 30.1% of aspiring health professionals had adequate health literacy. Differences between our findings, the findings of Löwe et al. (35) and those of previous studies may be attributable to heightened health awareness due to the COVID-19 pandemic. The timing of our study (December 2022) and that of Löwe et al. (35) (May–November 2022) may have captured a cohort more actively engaged in health topics. Supporting this, a study on the Generation Z reported increased health-conscious behavior following the pandemic (37). Other studies show that the health awareness of the general population has increased after the COVID-19 pandemic (38, 39). A possible selection bias may also explain the unexpectedly high health literacy levels observed in our survey. It is well-documented that individuals with higher education, better health, and a greater interest in health topics are more likely to participate in voluntary health surveys (40). Rational choice theory offers an explanation. It assumes that participants will only take part in a survey if the expected benefit (interest in the topic, monetary incentive, loyalty or social responsibility) is higher than the costs and effort involved (41).

Digital health literacy (60.9%) was also higher in our study than in other reports. For instance, Schaeffer et al. (42) found that only 24.4% of the German population had high digital health literacy. In two other studies on the digital health literacy of the German population, 52–76% showed limited digital health literacy (9, 16).

In this study, exploratory analyses identified some predictors for higher health literacy that showed no or not such strong correlations with health literacy in other studies. Notably, male gender was significantly associated with both higher general and digital health literacy – consistent with findings by Dadaczynski et al. (43), who reported lower digital health literacy among female students during the pandemic. However, this study surveyed students in general, i.e., not exclusively students with a health-related background.

In our study, additional predictors of digital health literacy included older age, greater self-efficacy, and higher alcohol consumption. Smoking showed a significant association only in univariable analyses. Rababah et al. (44) found that female students and non-smokers reported higher health literacy. Schultes (36) however, did not find any link between alcohol use and health literacy. Conversely, Ernstmann et al. (45) reported weak correlations between health literacy and substance use among younger people. Löwe et al. (35) found that master’s students with better sleep, a healthier diet, lower alcohol use, and higher life satisfaction had greater health literacy. The social-ecological model posits that health literacy’s impact on alcohol use is mediated by social norms, environmental factors, and healthcare system influences, shaping individuals’ drinking behaviors within broader social contexts (46). Thus, the evidence on the impact of alcohol and tobacco use on health literacy remains inconclusive and warrants further investigation.

Regarding the higher health literacy among master’s students, it is assumed that advanced study and life experience lead to higher health literacy. In general, students are in a critical phase of their lives in terms of health. The state of health is usually assessed as “good” or “very good,” but these values are not reflected in health literacy, especially among Bachelor’s students. Löwe et al. (35) recommend targeted promotion of health literacy early in academic training.

Many participants in our study reported difficulties understanding and locating health information, raising concerns about their ability to communicate such information to patients effectively. Ensuring excellent patient care and promoting self-care among future healthcare professionals requires a high degree of health literacy. In line with this, Scheffer et al. (42) recommend that not only general health literacy, but also professional health literacy should be further strengthened in the training and studies of healthcare professionals.

Professional health literacy refers to the health literacy of individuals working in the healthcare sector. Surprisingly, 55–70% of the nurses and doctors surveyed rated their skills as “quite positive,” depending on the area of professional health literacy (42).

### Limitations

The first limitation of this study is the composition of the study population (sampling bias). Lecturers in the therapy professions, midwifery, emergency medical services, nursing and medical-technical professions mainly carried out the recruitment of study participants. Therefore, most of the study participants were trainees and students at the vocational colleges and universities where the study personnel work.

The generalizability of the results is also limited by the fact that the sample composition does not accurately reflect the actual number of training places in Germany’s various healthcare professions. For example, physiotherapy was represented by a relatively high proportion of trainees and students (*n* = 187), whereas nursing was only represented by a tiny proportion (*n* = 83). According to statistics from the Federal Employment Agency for the 2021/2022 school year, a total of 107,385 people were in training for a nursing profession and 25,498 for physiotherapy as of 31.12.2021 (47). The generalizability of the results is also limited because the proportion of study participants per federal state was not distributed appropriately. Most participants (83%; 542/655) came from three large federal states in Germany (Hesse, Baden-Württemberg, North Rhine-Westphalia, see also Supplementary material 3).

Of the 880 participants who initiated the survey, 655 completed it, and 195 data sets were excluded (see reasons for exclusion in Figure 1). This proportion of excluded data sets (22%) represents a further limitation of this study. For example, a total of 77 participants did not (entirely) answer the questions on health literacy. The reasons for this could be that these questions were not understood, and there was no opportunity to ask, which is a typical limiting factor of online surveys. The exclusion of (incomplete) data sets in online surveys and drop-out rates up to 30% are not uncommon (48).

Standardized questionnaires were used to record general and digital health literacy, which have been used successfully for years in international studies involving several 1,000 individuals (28). The assessment of self-reported health literacy with questionnaires could be biased due to possible distortions in response behavior (recall bias, social desirability). For example, participants could overestimate or underestimate their own competencies.

We included more than 20 variables in the regression analyses. However, we did not assess skills and competencies in using (digital) technologies or artificial intelligence applications. These variables might have influenced the performance of the regression models or impacted the results of the regression analyses.

### Implications

This study highlights the need for further research. Further studies should be conducted on health literacy and digital health literacy among trainees and students of healthcare professions to be able to describe the level of health literacy in a larger and more representative sample even more accurately and reliably. In addition, further research should be conducted into which (modifiable) predictors or factors influence the level of health literacy in this target group. There are already initial findings on how health literacy can be taught to trainees and students in the healthcare professions (49). Further studies seem necessary to analyze which concepts and methods are most efficient in the German education system and in which form of teaching.

In Germany, schools and universities generally have overall responsibility for training in the healthcare professions, such as nursing and physiotherapy. Depending on the federal state, practical and theoretical instruction takes place at state-recognized vocational schools, clinics or healthcare schools and generally lasts 3 years of full-time training for all non-academic healthcare professions. In 2020, the Midwifery and Nursing Act was reformed, which not only introduced the 3-year generalized nursing training, but also the full academization of midwifery training. Nevertheless, the topic of health literacy has not yet been sufficiently addressed in the curricula for initial, further and continuing nursing training or in the current nursing-related degree courses (45).

Institutions that train healthcare professionals should therefore pay more attention to the promotion of health literacy in medical training programs when designing the curriculum. Ideally, a “health literacy” module could be implemented or the skills could be taught as part of longitudinal modules (49). For example, practice-oriented and interprofessional learning could be applied through simulation-based training in different modules, which would allow students to practice their communication and patient interaction skills. Furthermore, teachers should be enabled to use appropriate media skills in lessons, such as analysis of trigger videos or interaction videos with trained clients, in order to promote digital health literacy among trainees/students (49). In this context, the difficulties involved in finding and understanding (digital) information should be addressed explicitly through “teach back training” with trainees, for example. In the future, the aim should be to offer an academic professional education to all healthcare professionals in Germany, as an independent and entirely evidence-based teaching of health literacy can be conducted ideally through academic training with an analytical and conceptual approach.

### Conclusion

The results of this study show that trainees and students in the allied health professions in Germany have better general and digital health literacy than the general population or students in general. However, there is also a clear need to improve the health literacy of this target group, as only around 50% of participants have a “high” level of health literacy. Trainees in healthcare professions and students pursuing health-related degree courses require excellent health literacy to effectively communicate targeted knowledge to their patients and provide informed advice. To improve health literacy, it should be integrated more strongly into the training curricula.

## Data Availability

The datasets presented in this study can be found in online repositories. The names of the repository/repositories and accession number(s) can be found at: Mendeley Data Repository (doi: 10.17632/xhd86y2vwc.1) https://data.mendeley.com/datasets/xhd86y2vwc/1.

## Author’s note

A preliminary version of this manuscript was previously published as a preprint on Research Square (DOI: https://doi.org/10.21203/rs.3.rs-7633983/v1). The preprint allowed early access to the results and facilitated scientific exchange. The current version represents the peer-reviewed, revised, and updated article. The full reference is listed in the reference section (50).
