# Supplementary material for: Health literacy of vocational and university students in the allied health professions in Germany—a cross-sectional study
Source: Front Public Health. 2025 Dec 4;13:1711608. doi: 10.3389/fpubh.2025.1711608 (PMC12711698; doi:10.3389/fpubh.2025.1711608)
Supplement: Supplementary file 1 [file Data_Sheet_1.pdf]

Supplementary material 1: Variables (sociodemographic characteristics, educational situation, health and health-related behavior of the participants)

Variable/Item	Question (operationalization)	Answer/Response options	Source	Categorization for regression analyses
Age	How old are you?<br>Please enter your exact age in years.	Input field (metric)	None	- 18–21<br>- 22+
Gender	Which gender do you identify with?	- Male<br>- Female<br>- Diverse<br>- No answer	Braun 2019	- Male<br>- Female
Place of residence	In which federal state do you live?	- Baden-Württemberg<br>- Bavaria<br>- Berlin<br>- Brandenburg<br>- Bremen<br>- Hamburg<br>- Hesse<br>- Mecklenburg-West Pomerania<br>- Lower Saxony<br>- North Rhine-Westphalia<br>- Rhineland-Palatinate<br>- Saarland<br>- Saxony<br>- Saxony-Anhalt<br>- Schleswig-Holstein<br>- Thuringia<br>- Abroad	None	Not included
Migration background	Do you have a migration background?	- Yes<br>- No<br>- No answer	None	- Yes<br>- No
Socioeconomic status	How do you assess your own wealth?	- Low<br>- Medium	Domanska 2021	- Low<br>- Medium<br>- High

Variable/Item	Question (operationalization)	Answer/Response options	Source	Categorization for regression analyses
		- High<br>- No answer		
Highest educational degree	What is your highest educational degree?	- Lower secondary school (*Hauptschulabschluss*)<br>- Upper secondary school (*Realschulabschluss / Fachoberschulreife / mittlere Reife*)<br>- University of Applied Sciences entrance qualification (*Fachabitur / Fachhochschulreife*)<br>- University entrance qualification (*Abitur / allgemeine Hochschulreife*)<br>- Bachelor<br>- Master (*Master/Magister/Diplom*)<br>- Other (please report)<br>- No answer	GEDA-EHIS 2015 Item 143	- Low (Lower secondary school, Upper secondary school)<br>- High (University of Applied Sciences / University entrance qualification, Bachelor, Master)
Field of education (profession)	In which medical field is your education or training?	- Nursing<br>- Occupational therapy<br>- Physiotherapy<br>- Speech therapy<br>- Paramedics<br>- Midwifery<br>- medical-technical professions<br>- Other	None	- Nursing<br>- Occupational therapy<br>- Physiotherapy<br>- Speech therapy<br>- Paramedics<br>- Midwifery<br>- medical-technical professions
Degree sought	What degree are you pursuing?	- State examination (*Staatsexamen*)<br>- Bachelor<br>- Master	None	Not included

Variable/Item	Question (operationalization)	Answer/Response options	Source	Categorization for regression analyses
Prior medical education	Do you have completed medical education or degree?<br>If yes, have you worked in this profession longer than 2 years?	- Yes, less than 2 years<br>- Yes, more than 2 years<br>- No	None	- Yes < 2 years<br>- No
Type of education	What type of education are you currently in?	- Training (*Ausbildung*)<br>- Dual studies (*Duales Studium*)<br>- University studies (*Studium*)	None	- Training<br>- Dual studies<br>- University studies
Stage of education	In which year/semester of your education are you currently?	- 1st year<br>- 2nd year<br>- 3rd year<br>- 4th year or more<br>- 1st semester<br>- 2nd semester<br>- 3rd semester<br>- 4th semester<br>- 5th semester<br>- 6th semester<br>- 7th semester<br>- 8th semester<br>- 9th semester or more	None	- 1st year / 1-2 semesters<br>- 2nd year / 3-4 semesters<br>- 3+ years / 5+ semesters
Mindfulness towards own health	How much do you generally pay attention to your health?	- Very much<br>- Much<br>- Moderate<br>- Less<br>- Not at all	GEDA-EHIS 2015 item 134	- Low (not at all, less, moderate)<br>- High (much, very much)
Self-assessment of health status	How would you rate your health?<br>Please give a number between 0 (worst possible health) and 100 (best possible health).	Input field (metric)	EQ-5D	- Low (0-75)<br>- High (76-100)
Physical activity	How often do you exercise?	- Never<br>- 1x/month<br>- 1x/week<br>- 2-3x/week	None	- Never/rare (never, 1x/month)<br>- Regular (1x/week)

Variable/Item	Question (operationalization)	Answer/Response options	Source	Categorization for regression analyses
		- 4-5x/week<br>- daily		- Frequent (2x/week to daily)
Physical activity intention	Do you intend to exercise more often than before?	- Yes<br>- No<br>- No answer	None	Not included
Diet	How often do you consciously eat healthily during the week?	- Rarely<br>- 1x/week<br>- 2-3x/week<br>- Every day<br>- No answer	GEDA-EHIS 2015 item 106	- Poor (rarely, 1x/week)<br>- Good (2x/week to daily)
Barriers to healthy diet	What prevents you from eating healthy every day?	- Lack of time<br>- Healthy food is too expensive<br>- Prefer unhealthy food<br>- Lack of knowledge on healthy eating<br>- I already eat healthy<br>[Multiple answers possible]	GEDA-EHIS 2015 item 107	Not included
Tobacco consumption	Do you smoke?	- Yes, daily<br>- Yes, occasionally<br>- No, not anymore<br>- Never smoked<br>- No answer	GEDA-EHIS 2015 item 112	- Yes (daily, occasionally)<br>- No (not anymore, never)
Alcohol consumption	How often do you drink alcohol?	- Daily<br>- Several times a week<br>- Once a week<br>- At least once a month<br>- I do not drink alcohol<br>- No answer	Adapted from GEDA-EHIS 2015 item 121	- High (daily, several times a week)<br>- Low/no (once a week, once a month, never)
Hypertension	Have you ever been diagnosed with high blood pressure (hypertension)?	- Yes<br>- No<br>- I don't know<br>- No answer	Adapted from GEDA-EHIS 2015 item 9	- Yes<br>- No

Variable/Item	Question (operationalization)	Answer/Response options	Source	Categorization for regression analyses
Chronic diseases	Do you have a chronic disease or long-standing health problem (persisting for more than 6 months or expected to persist)?	- Yes<br>- No<br>- No answer	GEDA 2015 item 5	- Yes<br>- No
Mental health disorders	Have you ever been diagnosed with a mental health disorder?	- Yes<br>- No<br>- No answer	None	- Yes<br>- No
Medication use	Do you regularly take medications (at least 3 times per month)?	- Yes<br>- No<br>- No answer	GEDA-EHIS<br><br>2015 item 59	- Yes<br>- No
Types of medication	If yes, which medications do you regularly take?	- Painkillers<br>- Sleeping pills<br>- Thyroid medications<br>- Blood pressure / arrhythmia medications<br>- Antihistamines<br>- Corticosteroids<br>- Beta-2 sympathomimetics / anticholinergics<br>- Diuretics<br>- Birth control pills<br>- Others	None	Not included
Quality of life	How do you rate your current quality of life?	Visual analog scale with smileys:<br>- Very bad<br>- Bad<br>- Sufficent<br>- Satisfactory<br>- Good<br>- Very good	Based on EQ-5D	- Low (very bad to satisfactory)<br>- High (good to very good)
Social support	How many people do you have close enough to rely on for help with serious personal problems?	- None<br>- 1-2<br>- 3-5<br>- 6 or more	GEDA-EHIS 2015 item 127	Not included

Variable/Item	Question (operationalization)	Answer/Response options	Source	Categorization for regression analyses
Self-efficacy (general situation)	The following statements may apply to you to a greater or lesser extent: I can rely on my abilities in difficult situations. (Likert scale 0-4)	- does not apply at all (0)<br>- applies to a small extent (1)<br>- applies somewhat (2)<br>- applies fairly well (3)<br>- applies completely (4)	GEDA-EHIS 2015 item 133	"Self-efficacy total score (0-12 points)" consists of 3 questions (0-4 points each):<br>- low (0-9 points)<br>- high (10-12 points)
Self-efficacy (problems)	The following statements may apply to you to a greater or lesser extent: I can solve most problems by myself. (Likert scale 0-4)			
Self-efficacy (tasks)	The following statements may apply to you to a greater or lesser extent: I can manage even difficult and complicated tasks well. (Likert scale 0-4)			
Health literacy and healthcare staff	Health literacy is defined as the skills to find, understand, evaluate and apply health information to maintain/improve health and quality of life. How important is it that healthcare professionals have good health literacy? (Scale 0-10)	Numeric scale from 0 (not important) to 10 (very important)	None	Not included

References:

- Braun et al. (2019): Cross-cultural adaptation, internal consistency, test-retest reliability and feasibility of the German version of the evidence-based practice inventory. BMC Health Serv Res 19 (1), 455. DOI: 10.1186/s12913-019-4273-0.
- Devlin et al. (2017): EQ-5D and the EuroQol Group: Past, Present and Future. Applied health economics and health policy 15 (2), 127-137. DOI: 10.1007/s40258-017-0310-5.
- Domanska et al. (2021): Gesundheitskompetenz und Gesundheitsverhalten im Jugendalter: Ergebnisse einer bundesweiten Online-Befragung Jugendlicher. Präv Gesundheitsf., 1-9. DOI: 10.1007/s11553-021-00913-1.
- Robert Koch-Institut (2017): Fragebogen zur Studie „Gesundheit in Deutschland aktuell“ GEDA 2014/2015-EHIS.
