# Supplementary material for: Health literacy of vocational and university students in the allied health professions in Germany—a cross-sectional study
Source: Front Public Health. 2025 Dec 4;13:1711608. doi: 10.3389/fpubh.2025.1711608 (PMC12711698; doi:10.3389/fpubh.2025.1711608)
Supplement: Supplementary file 2 [file Data_Sheet_2.pdf]

## Supplementary material 2: Additional information on the characteristics and health behavior of the participants (n = 655)

| | | Number (n) | Percent (%) |
|---|---|---|---|
| Intention to increase physical activity (n = 643) | | | |
| | Yes | 456 | 70.9 |
| | No | 187 | 29.1 |
| What prevents you from eating healthy every day? (Multiple answers possible; n = 655) | | | |
| | Lack of time | 387 | 59.1 |
| | Healthy food is too expensive | 189 | 28.9 |
| | Prefer unhealthy food | 109 | 16.6 |
| | Lack of knowledge on healthy eating | 65 | 9.9 |
| | I already eat healthy | 158 | 24.1 |
| | Not answer | 47 | 7.2 |
| Hypertension (n = 652) | | | |
| | Yes | 36 | 5.5 |
| | No | 588 | 90.2 |
| | I do not know | 28 | 4.3 |
| What medications do you take regularly? (Multiple answers possible; n = 655) | | | |
| | Painkillers | 95 | 14.5 |
| | Antidepressants | 45 | 6.9 |
| | Sleeping pills | 15 | 2.3 |
| | Thyroid medications | 36 | 5.5 |
| | Blood pressure / arrhythmia medications | 20 | 3.1 |
| | Antihistamines | 16 | 2.4 |
| | Corticosteroids | 8 | 1.2 |
| | Beta-2 sympathomimetics / anticholinergics | 7 | 1.1 |
| | Diuretics | 1 | 0.2 |
| | Birth control pills | 137 | 20.9 |
| | Other | 67 | 10.2 |
| | No answer | 344 | 52.5 |
