# Supplementary material for: Health literacy of vocational and university students in the allied health professions in Germany—a cross-sectional study
Source: Front Public Health. 2025 Dec 4;13:1711608. doi: 10.3389/fpubh.2025.1711608 (PMC12711698; doi:10.3389/fpubh.2025.1711608)
Supplement: Supplementary file 3 [file Data_Sheet_3.pdf]

Supplementary material 3: Distribution of participants across the 16 federal states in Germany

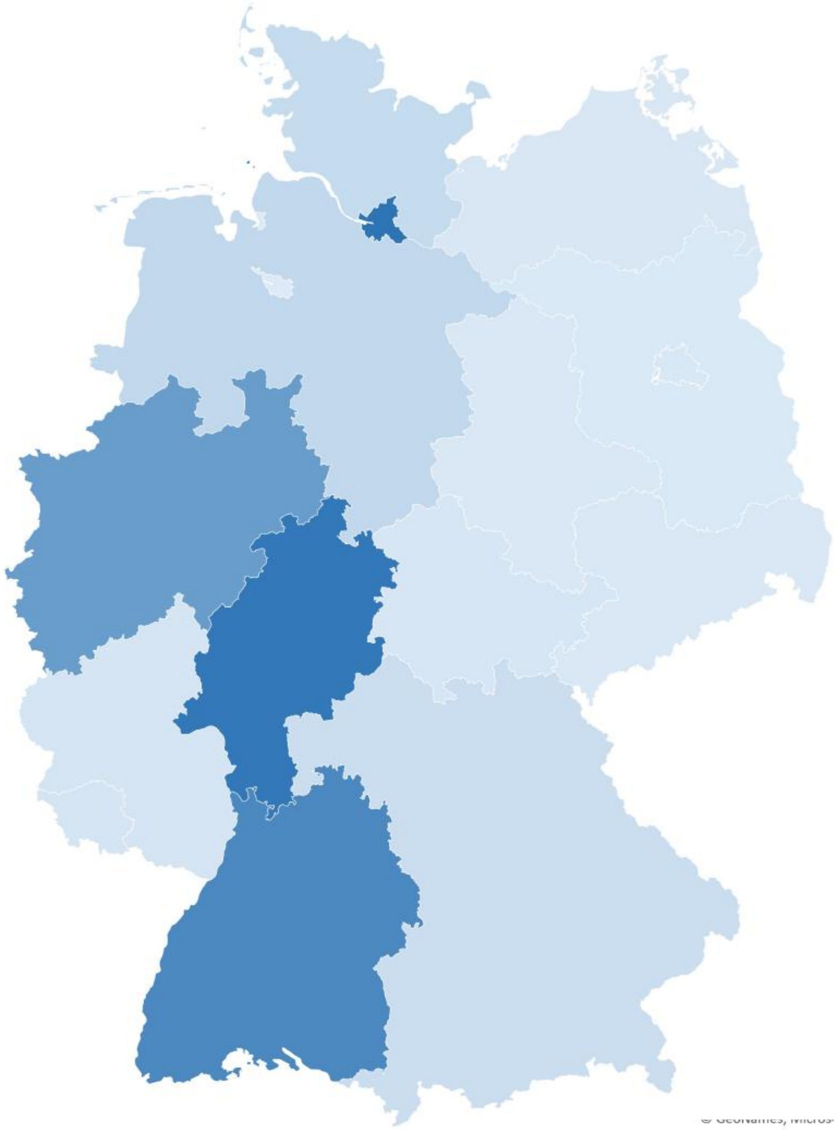

Federal state	Residents	n	Percent
Hanseatic City of Hamburg	1.787.408	36	0,00201
Hesse	3.520.031	121	0,00196
Baden-Württemberg	2.858.714	182	0,00167
North Rhine-Westphalia	17.865.516	239	0,00134
Lower Saxony	7.926.599	25	0,00032
Schleswig-Holstein	671.489	8	0,00028
Bavaria	10.879.618	28	0,00022
Saarland	4.052.803	1	0,00010
Rhineland-Palatinate	2.170.714	4	0,00010
Thuringia	12.843.514	2	0,00009
Mecklenburg-Western Pomerania	4.084.851	1	0,00006
Berlin	2.484.826	2	0,00006
Saxony	6.176.172	2	0,00005
Saxony-Anhalt	2.245.470	1	0,00004
Brandenburg	1.612.362	1	0,00004
Hanseatic City of Bremen	995.597	0	0,00000

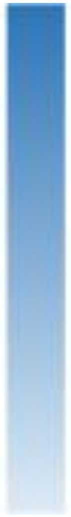

Number of participants (n = 655; 2 of whom reside abroad) by federal state (n = 16). The colors indicate the ratio between the number of study participants and the number of inhabitants in each federal state, with dark and light shades of blue indicating higher and lower rates, respectively. For example, Hamburg, with 36 participants and 1.787 million inhabitants, has the highest rate (0.00201%), and Bremen, with 0 participants, has the lowest rate (0%). (Data source: Federal Statistical Office; www.destatis.de; “Federal states with capitals by area, population, and population density as of December 31, 2016”).
