# Supplementary material for: Health literacy of vocational and university students in the allied health professions in Germany—a cross-sectional study
Source: Front Public Health. 2025 Dec 4;13:1711608. doi: 10.3389/fpubh.2025.1711608 (PMC12711698; doi:10.3389/fpubh.2025.1711608)

Supplementary material 4: Response behavior (absolute and percentage) of participants (n = 655) for the 16 individual items of the HLS-EU-Q16-DE (general health literacy)

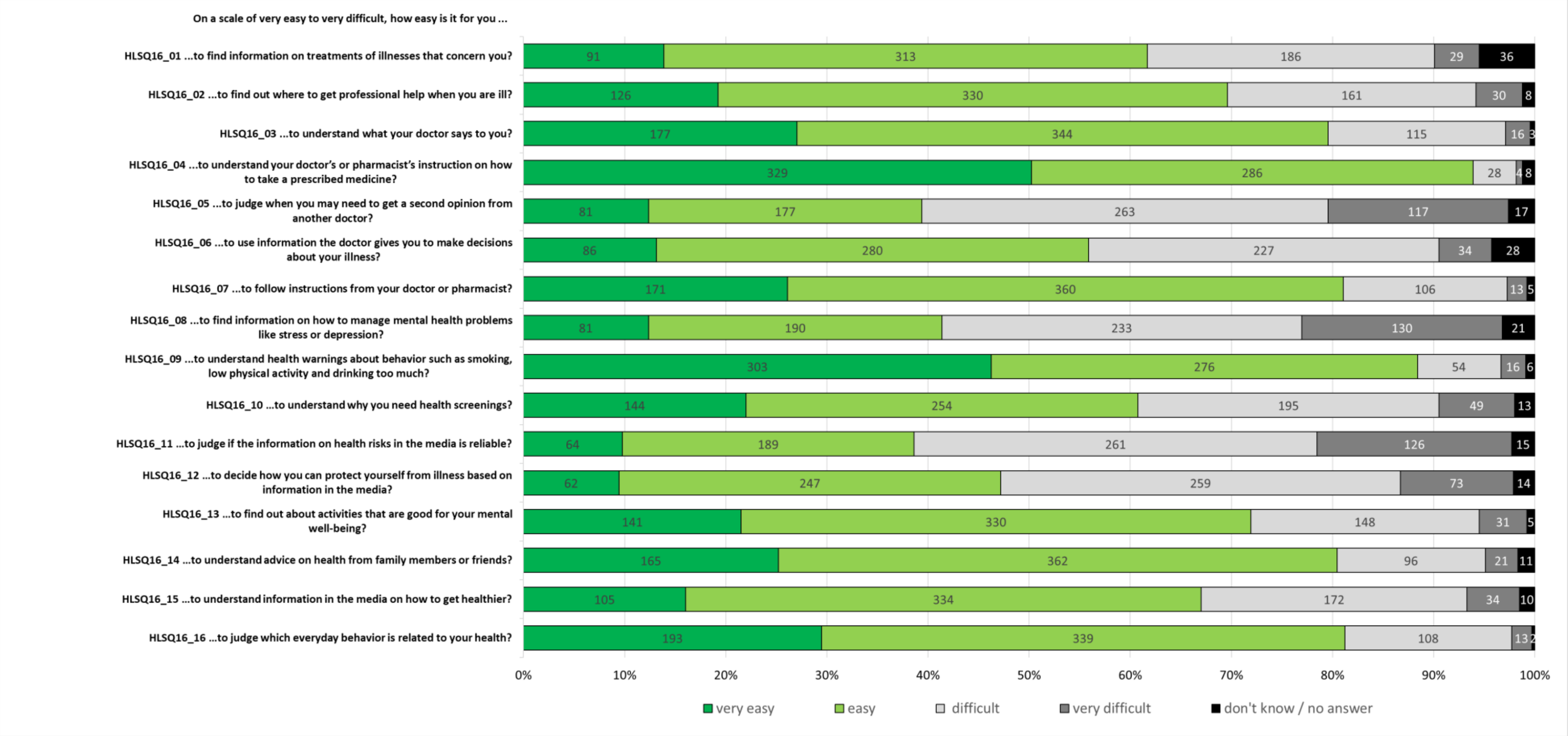

Supplement: Supplementary file 4 [file Data_Sheet_4.pdf]
