# Supplementary material for: Health literacy of vocational and university students in the allied health professions in Germany—a cross-sectional study
Source: Front Public Health. 2025 Dec 4;13:1711608. doi: 10.3389/fpubh.2025.1711608 (PMC12711698; doi:10.3389/fpubh.2025.1711608)

Supplementary material 5: Response behavior (absolute and percentage) of participants (n = 600) for the 16 individual items of the HLS19-DIGI-DE (digital health literacy)

HLS19-DIGI-DE Item 1.1: How many days in a typical week do you use health-related websites? Would you say...

HLS19-DIGI-DE Item 1.2: And how many days in a typical week do you use social media (including online forums) to learn about or discuss health topics? Would you say...

HLS19-DIGI-DE Item 1.3: How many days in a typical week do you use digital devices related to health or medical care, e.g., pedometer, smartwatch, fitness tracker, etc.?

HLS19-DIGI-DE Item 1.4: Health apps on mobile phones [Interviewer note: e.g., to calculate calorie consumption, support medication intake, measure physical activity, promote healthy sleep, etc.]

HLS19-DIGI-DE Item 1.5: Digital interaction with healthcare providers [Interviewer note: e.g., online appointment scheduling, accessing personal health records, electronic delivery/transmission of medical tests, communicating with a provider, answering questions about a healthcare service, etc.]

HLS19-DIGI-DE Item 1.6: Finally, how many days in a typical week do you use other digital health information services? Would you say...

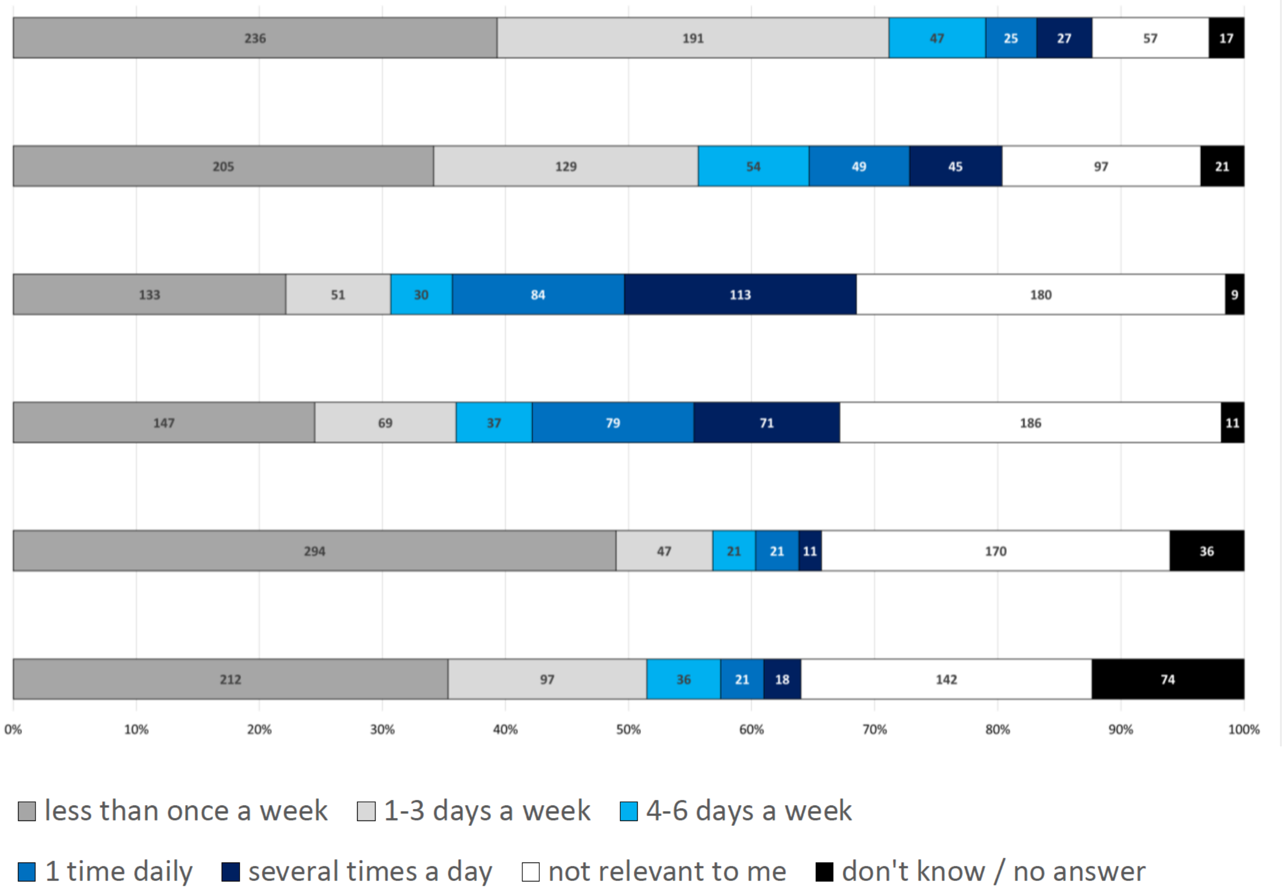

When you search for health information online, how easy or difficult is it for you...

HLS19-DIGI-DE Item 2.1: … use the proper words or search query to find the information you are looking for?

HLS19-DIGI-DE Item 2.2: … find the exact information you are looking for?

HLS19-DIGI-DE Item 2.3: … to understand the information found?

HLS19-DIGI-DE Item 2.4: … decide whether the information is reliable or not?

HLS19-DIGI-DE Item 2.5: … decide whether the information is written with commercial interests (eg, by people trying to sell a product)?

HLS19-DIGI-DE Item 2.6: …check different websites to see whether they provide the same information?

HLS19-DIGI-DE Item 2.7: … Decide if the information you found is applicable to you?

HLS19-DIGI-DE Item 2.8: … Use the information you found to make decisions about your health?

HLS19-DIGI-DE Item 3.1: How easy or difficult is it for you to clearly formulate your written message when communicating with a health provider?

HLS19-DIGI-DE Item 3.2: How easy or difficult is it for you to express your opinion, thoughts, or feelings, or ask a question in writing on social media including online forums?

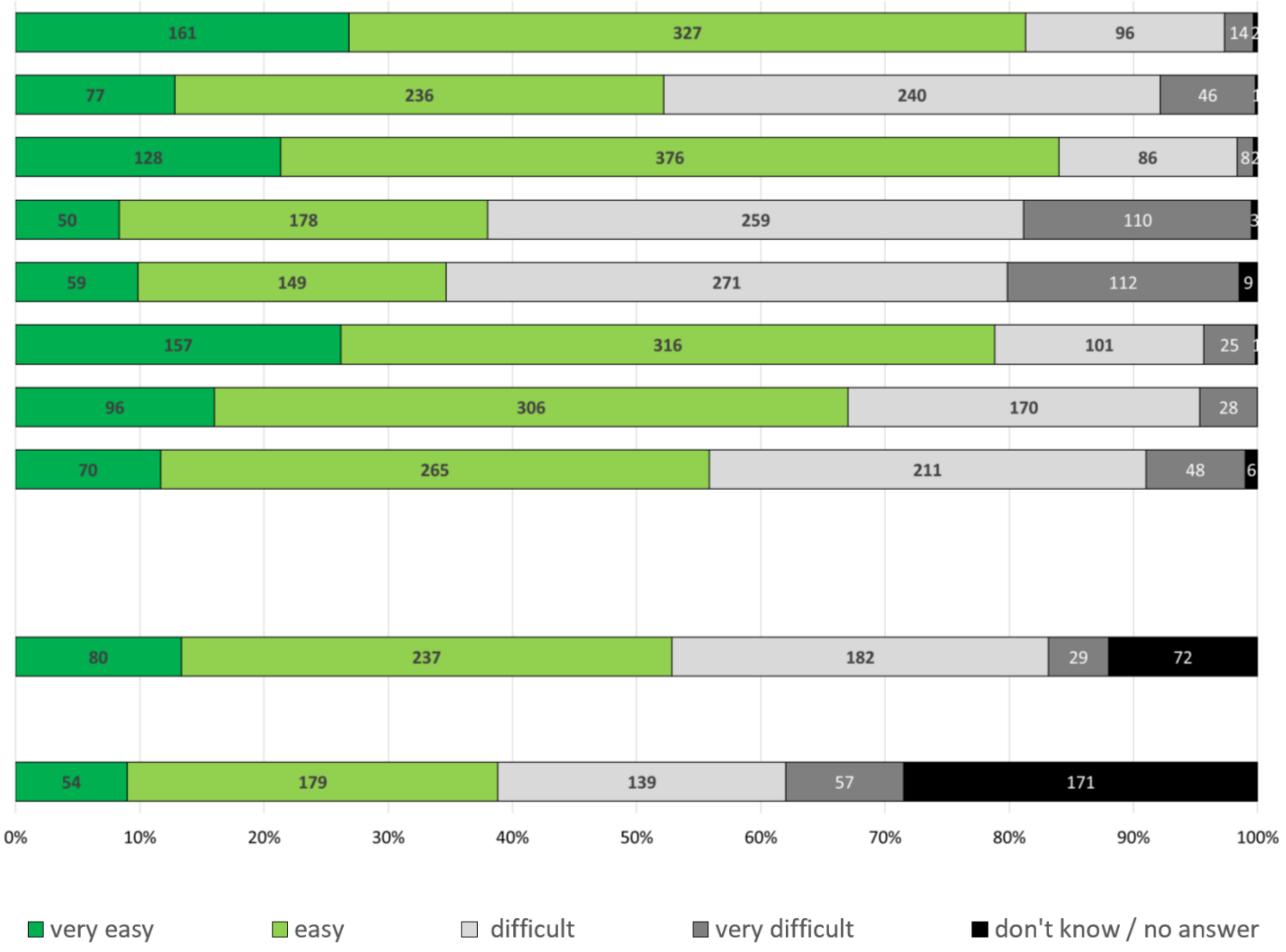

Supplement: Supplementary file 5 [file Data_Sheet_5.pdf]
